# The Structural and Molecular Underpinnings of Gametogenesis in *Toxoplasma gondii*

**DOI:** 10.3389/fcimb.2020.608291

**Published:** 2020-12-07

**Authors:** Ramiro Tomasina, María E. Francia

**Affiliations:** ^1^ Laboratory of Apicomplexan Biology, Institut Pasteur de Montevideo, Montevideo, Uruguay; ^2^ Department of Parasitology and Mycology, School of Medicine, Universidad de la Republica, Montevideo, Uruguay

**Keywords:** *Toxoplasma gondii*, sexual cycle, molecular cues, sexual differentiation, parasite dissemination

## Abstract

*Toxoplasma gondii* is a widely prevalent protozoan parasite member of the phylum Apicomplexa. It causes disease in humans with clinical outcomes ranging from an asymptomatic manifestation to eye disease to reproductive failure and neurological symptoms. In farm animals, and particularly in sheep, toxoplasmosis costs the industry millions by profoundly affecting their reproductive potential. As do all the parasites in the phylum, *T. gondii* parasites go through sexual and asexual replication in the context of an heteroxenic life cycle involving members of the Felidae family and any warm-blooded vertebrate as definitive and intermediate hosts, respectively. During sexual replication, merozoites differentiate into female and male gametes; their combination gives rise to a zygotes which evolve into sporozoites that encyst and are shed in cat’s feces as environmentally resistant oocysts. During zygote formation *T. gondii* parasites are diploid providing the parasite with a window of opportunity for genetic admixture making this a key step in the generation of genetic diversity. In addition, oocyst formation and shedding are central to dissemination and environmental contamination with infectious parasite forms. In this minireview we summarize the current state of the art on the process of gametogenesis. We discuss the unique structures of macro and microgametes, an insight acquired through classical techniques, as well as the more recently attained molecular understanding of the routes leading up to these life forms by *in vitro* and *in vivo* systems. We pose a number of unanswered questions and discuss these in the context of the latest findings on molecular cues mediating stage switching, and the implication for the field of newly available *in vitro* tools.

## Introduction

Infecting about a third of the world’s population *Toxoplasma gondii* is a widely prevalent protozoan parasite, member of the phylum Apicomplexa, and the etiological agent of toxoplasmosis (Dubey, 2013; Torgerson and Mastroiacovo, 2013; Sherri et al., 2017). Its success as a pathogen is partly owed to its proficiency in invading virtually any nucleated cell in an immense range of warm-blooded hosts, ranging from humans and farm animals, to birds and marine species. Its main mechanism of pathogenesis is a consequence of its obligate intracellular lifestyle, which inevitably culminates in the lysis of the infected cell as the parasite replicates and expands. Notwithstanding, its ability to persist without killing the host is owed to its ability to switch from a fast dividing, disease-causing form, known as the tachyzoite, to a slow dividing, encysted/latent form, known as the bradyzoite. While the former is responsible for acute toxoplasmosis, the latter can persist chronically in its host. Acute toxoplasmosis most notably causes reproductive failure in humans, but it can also cause a range of clinical manifestations from eye to neurological disease in chronically infected immunocompromised individuals. Ocular toxoplasmosis is also frequent in congenitally or chronically infected immunocompetent individuals (Commodaro et al., 2009). In farm animals, and particularly in sheep, toxoplasmosis costs the industry millions by affecting reproductive outcomes (Dubey et al., 2020). Likewise, toxoplasmosis has been shown to greatly affect sensitive populations of wild animals, with particular effects on marine life and devastating ecological outcomes (Shapiro et al., 2019). A common route of infection for humans and other carnivores is the consumption of undercooked meat infected with bradyzoites lodged as cysts in skeletal muscle or the brain (Pinto-Ferreira et al., 2019). Vertical transmission is also possible when the infection is acquired by a naive host during pregnancy. Transmission is also possible by the serendipitous interaction with infective oocysts, shed by feline species in their feces.

Felines are *T. gondii*’s definitive host, whereby the parasite undergoes its sexual cycle. Sexual differentiation entails the formation of macro and a microgametes, and zygote formation can occur upon their combination. This process is key to the generation of genetic diversity as this is the only stage at which genetic admixing can occur as any other zoite life stage of this parasite is haploid. Equally important, this process is central to dissemination, as a single felid can shed hundreds of millions of oocysts, and these are stable in the environment for at least a year. The annual oocyst burden has been estimated to range from 3 to 434 oocysts per square foot in different community surveys in the United States. Oocyst burden is concentrated in areas where cats preferably defecate, this is places with loose soil, such as gardens, children’s play areas, and sandboxes (Torrey and Yolken, 2013). An astonishing example of how potentially harmful oocysts can be in the environment is showcased by the toxoplasmosis epidemic in sea otters in coastal California. Infectious oocysts shed by domestic cats, located many miles away, are dragged along by the river that flows into the otter’s natural aquatic habitat (Miller et al., 2002; Conrad et al., 2005; Shapiro et al., 2012; Vanwormer et al., 2013). Close to 9% of their mortality can be ascribed to meningoencephalitis caused by *Toxoplasma* (Thomas and Cole, 1996; Shapiro et al., 2019).

Understanding the biology of the life forms that precede the formation of infective oocyst, is, in addition to a fascinating ill-understood source of biological data, central to developing efficient intervention strategies to prevent their formation and spread. Here, we review the current state of the art on gametogenesis, integrating the plethora of fantastic structural insight acquired through classical microscopy with exciting new molecular insights provided by *in vitro* and *in vivo* systems on the molecular cues facilitating stage switching.

## The Life Stages of *Toxoplasma gondii*

The life forms of *T. gondii* can be generally grouped in two; the ones that replicate clonally and those that are generated by combination of gametes.

The asexually or clonally replicating forms develop in warm-blooded intermediate hosts whereby two distinct forms can be identified; the tachyzoite and the bradyzoite. The tachyzoite is a highly proliferative form commonly associated with acute infection, reactivation, and vertical transmission. The bradyzoite, on the other hand, is a latent, albeit metabolically active, slow growing encysted form. Bradyzoites are commonly associated with the chronic stages of toxoplasmosis, persistence, immune evasion, and are refractory to currently available anti-toxoplasmosis pharmacotherapies (Weiss and Kim, 2000; Lyons et al., 2002; Dzierszinski et al., 2004; Sullivan and Jeffers, 2012; Knoll et al., 2013; Watts et al., 2015; Sinai et al., 2017; Cerutti et al., 2020). Evasion is mediated by cell type tropism and sequestration to immune-privileged sites such as the brain and skeletal muscle, as well as the process of encystation (Sullivan and Jeffers, 2012; Knoll et al., 2013). Both tachyzoites and bradyzoites follow a cell division scheme known as endodyogeny consisting of a single round of DNA replication by semi-closed nuclear mitosis. The internal assembly of two daughter cells occurs concomitantly with nuclear mitosis, inside the mother cell (Francia and Striepen, 2014; White and Suvorova, 2018; Gubbels et al., 2020). Tachyzoite cell division is rapid, generating two new cells per mother cell every 6–8 h. Bradyzoites instead divide slower but can assemble and sustain between 1000–2000 bradyzoites per cyst (Weiss and Kim, 2000; Knoll et al., 2013).

**Figure 1:**
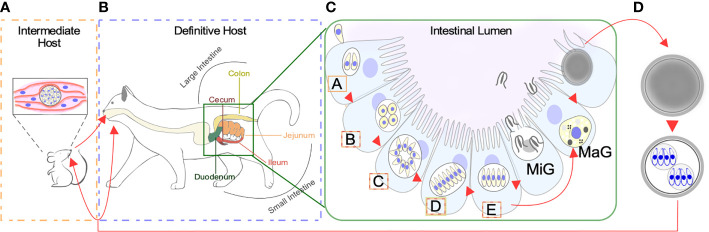
Life Stages of *Toxoplasma gondii*. **(A)** Warm blooded animals can act as intermediate hosts. Slow dividing bradyzoites can persist both in the brain and skeletal muscle within a tissue cyst. Transmission of bradyzoites can occur through carnivorism. Intermediate hosts can be infected by sporulated oocyst contaminating the environment. **(B)** Species from the Felidae family act as definitive hosts. Infection can occur by consuming tissue cysts containing bradyzoites, or sporozoites contained within sporulated oocysts. Differentiation of bradyzoites into asexually expanding merozoites happens along the intestinal tract. **(C)** Bradyzoites will sequentially differentiate into A-E merozoites prior to giving rise to macro (MaG) and micro (MiG) gametes. The intestinal segment where each meront is found more often is color coded according to B. **(D)** Combination of MaG and MiG gives rise to a unsporulated oocysts which mature in the environment becoming sporulated, sporozoite containing, infective oocysts.

When a feline eats undercooked meat infected with a cyst containing bradyzoites, most commonly from an infected mouse, the parasite can gain access to the definitive host’s gastrointestinal tract [**Figure 1A** (Dubey, 2006)]. Here, it can develop into either one of two life-forms: the tachyzoite or a merozoite. As in other hosts, tachyzoites can disseminate throughout the feline body and switch to bradyzoite residing in immune-privileged sites. However, clinical signs of toxoplasmosis are rarely observed in cats. The disease is more likely to occur in cats with suppressed immune systems, including young kittens and cats with feline leukemia virus (FeLV) or feline immunodeficiency virus (FIV). Toxoplasmosis is more severe in transplacentally infected kittens which can develop a variety of clinical manifestations ranging from hepatitis to cholangiohepatitis, pneumonia, and encephalitis (Dubey and Carpenter, 1993a; Dubey and Carpenter, 1993b; Webb et al., 2005; Calero-Berna and Gennari, 2019). More frequently, however, bradyzoites will differentiate into merozoites within the feline enterocytes (**Figures 1B, C**) (Frenkel, 1973; Dubey et al., 1998; Weiss and Kim, 2000; Webb et al., 2005; Dubey, 2009; Dubey, 2013). This transformation initiates with several rounds of rapid asexual expansion within the intestinal epithelium as merozoites, initiating the sexual replication track.

While tachyzoites invade and replicate extra-intestinally, merozoites develop only within enterocytes. Recent work by Hehl and col. identified important gene expression differences between tachyzoites and merozoites that could underlie this cell type tropism. A subset of well characterized dense-granule (GRA), microneme (MIC), and rhoptry (ROP) genes, known to be involved in attachment, invasion, and host cell modification to allow intracellular replication, are exclusively expressed in tachyzoites, and notably downregulated in merozoites. Merozoites seem to preferentially express a large subset of surface membrane proteins, known as SAG1-related sequences (SRS) for their homology to the surface antigen protein TgSag1, making them enterocyte-interaction competent (Speer and Dubey, 1998; Ferguson, 2004; Hehl et al., 2015). This is consistent with findings in *Plasmodium* whereby gamete-gamete interaction, recognition, and fertilization is mediated by a family of proteins, known as the Pfs-230-related 6-Cys adhesins, characterized by the presence of the SRS fold (Arredondo and Kappe, 2017). In addition, SRS proteins have been proposed to stimulate immune responses stimulating molecules, playing a role in intestinal inflammation and diarrhea, thereby potentially contributing to the dispersal of oocysts from felines (Hehl et al., 2015).

## Structural Aspects of *T. gondii* Gametes and Pre-Gamete Stages

Only 2 days after tissue cysts are ingested by the cat, micro and macrogametes are formed initiating the sexual cycle (Dubey et al., 1998). Our knowledge on the ultrastructure of pre-sexual stages is set on outstanding electron microscopy studies done in the 60’s and 70’s (Colley and Zaman, 1970; Melton and Sheffield, 1970; Pelster and Piekarski, 1971; Frenkel and Dubey, 1972; Sinden et al., 1976). Work by Dubey & Frankel systematically recorded the morphological details of five pre-gamete stages, named A to E, sequentially formed upon colonization of the epithelial cells of the small and large cat’s intestine (Dubey and Frenkel, 1972) (**Figures 1B, C**). Of these, only D or E meronts may differentiate into sexual gametes. Type A merozoites are readily observed, 12–18 h post infection. Their shape is round and they are commonly found singly or as a collection of 2 or 3. A meronts are commonly found in the jejunum, at the surface of the epithelium and in the lamina propria. How these meronts replicate exactly, remains elusive (Dubey and Frenkel, 1972). Type B meronts are observable 12–54 h post infection, are ovoid in shape, and commonly found in the jejunum, the ileum, at the surface of the epithelium (near the base of villi) and in the lamina propria, and more rarely in the glandular epithelium. B meronts replicate by endodyogeny and endopolygeny (Dubey and Frenkel, 1972). C meronts are observable 24–54 h post infection. Their shape is elongated, and they are found at the same locations as B meronts. C meronts are thought to replicate by schizogony following a rosette pattern (Dubey and Frenkel, 1972). Type D meronts are observable between 40 h and 15 days after infection. Their shape is elongated, and they are commonly found in the jejunum, ileum and the colon (Dubey et al., 1998). Type D meronts can be further classified in three subtypes; subtype I, found 48–72 h post infection, divides by endopolygeny and is found in groups of 2-4. Subtype II is found 3–15 days post infection, divides by schizogony and is found in groups of 5-35 merozoites. Subtype III merozoites albeit ill-defined, are thought to arise from “splitting”; a sort of modified schizogony whereby a large uninucleated cytosolic mass parcels into single meronts (Dubey and Frenkel, 1972). Finally, type E meronts appear 3–15 days after infection. Their shape is elongated, and they are commonly found in the jejunum, ileum, and seldomly in the colon. They replicate by schizogony (Dubey and Frenkel, 1972).

Upon asexual differentiation of meront type (A-E), gametogenesis is preceded by the formation of macro and micro gamonts. Gamonts are detectable 3–15 days after experimental inoculation of kittens with infective oocyst (Dubey et al., 1998). Gamonts are found throughout the small intestine, especially in the ileum. The precursor of the male gametocyte, the microgamont, is of ovoid shape and represents 2%–4% of the mature gametocyte population. Microgamonts can yield 6-21 microgametes, with a mean of 12. They are ellipsoidal, with the nucleus occupying much of the cell body. The anterior end is a pointed structure called the perforatorium, within which reside two microtubule organizing centers (MTOCs) acting as basal bodies organizing two posterior flagella (Dubey et al., 1998; Ferguson and Dubremetz, 2013; Francia et al., 2016; Morlon-guyot et al., 2017). On the other hand, macrogamonts are subspherical containing a single nucleus. Each macrogamont will give rise to a single macrogamete (Dubey and Frenkel, 1972). The cytosol of macrogamonts is populated with protein-rich wall forming bodies, polysaccharide granules and lipid droplets, which will all contribute to wall formation by sequentially releasing their content during oocyst maturation (Ferguson et al., 1975; Freppel et al., 2019). The oocyst wall starts forming within the host cell before the macrogamete is released, implying that fertilization has to take place inside the host cell (Freppel et al., 2019).

Macro and microgamete formation are not equilibrated in output. Every round of gametogenesis produces 1 macrogamete, and 15–30 microgametes. The number of macrogametes exceeds the number of microgametes by an average of 19-fold (Ferguson, 2002). Given that *T. gondii* can efficiently proliferate asexually, it has been proposed that sexual proliferation only provides a real advantage when the possibility of producing genetic diversity between different strains infecting the same feline exists. When a single strain is involved in this process, zygote formation based on fertilization has been argued as an extremely inefficient process (Ferguson, 2002). Quantification of the relative frequencies obtained from genetic crosses between two parental strains with distinct drug resistance phenotypes, suggest that sexual differentiation yields Mendelian inheritance patterns in the progeny, arguing that oocysts are only formed by fecundation (Pfefferkorn and Pfefferkorn, 1980). However, the only physical evidence supporting fertilization that has been generated, to our knowledge, is an electron micrograph showing several microgametes attached to an extracellular macrogamete, taken by Ferguson [Figure 4 in (Ferguson, 2002)]. Based upon this, Ferguson proposed that the ample efficiency with which oocyst are shed could be explained by the formation of oocysts from macrogametes alone through parthenogenesis (Ferguson, 2002). Though this hypothesis has not yet been experimentally tested, the unbalanced output of gametogenesis together with the long journey that every microgamete has to do to fertilize one macrogamete, has complicated the visualization of the process and its definitive understanding. Hence, how, when and where fertilization happens in the gut of the felids is still to be deciphered (Ferguson and Dubremetz, 2013).

Unsporulated oocyst are shed by cat in their feces (**Figure 1D**). These sporulate 1–5 days after shedding, forming a mature infection-competent oocyst. Sporulation is temperature-dependent; it is optimally efficient at temperatures closer to 25°C; delayed at temperatures lower than 15°C and inhibited at 37 or 4°C. Presence of oxygen is also required for oocyst sporulation and infectivity (Dubey et al., 1970). Each sporulated oocyst contains two sporocyst with four sporozoites each (Dubey et al., 1970). This stage is equipped with a multilayer wall that protects it from adverse environmental conditions, this being the only stage of the whole cycle of this parasite that develops outside the host cell (Ferguson and Dubremetz, 2013) (Ferguson et al., 1975).

## Molecular and Structural Links Between Asexual and Sexual Replication

### From Cell Division Control to Orchestrating Motility: The Microtubule Organizing Centers of *T. gondii* in the Context of Microgamete Formation

*Toxoplasma gondii* assembles two flagella per cell only when differentiated to microgamete. For fertilization to take place, every microgamete leaves the host cell where it was generated, swims across the intestinal lumen, finds a host cell containing a macrogamete, crosses the cell membrane, and fecundates the macrogamete (Ferguson, 2002). Because fertilization of a macrogamete requires the microgamete to be able to move, the flagella play a pivotal role in licensing this step. Ultimately, if oocyst formation relies on fertilization, then microgamete mobility is paramount to *T. gondii’s* environmental dissemination.

The flagellar axoneme consists of 9 microtubule doublets and a central pair of microtubules, and it is nucleated from a centriole-based basal body, formed by two microtubule-based barrels known as centrioles (Dubremetz, 1971; Dubremetz, 1973; Morrissette and Sibley, 2002a; Morrissette and Sibley, 2002b; Francia et al., 2016). In related coccidian species, such as *Eimeria* and *Sarcocystis*, basal body structure has been proposed to consist of either 9 + 0 or 9 + 2 singlets microtubules, or a canonical basal body, akin to what is observed in metazoan, formed by 9 + 0 triplet microtubules. The exact basal body structure of *T. gondii* remains unclear. However, electron micrographs suggest that it could consist of a 9 + 1 singlet microtubule structure (Pelster and Piekarski, 1971; Scholtyseck et al., 1972; Vetterling et al., 1973; Ferguson et al., 1974; Mehlhorn and Heydorn, 1979; Mueller et al., 1981; Morrissette and Sibley, 2002a; Walker et al., 2013; Francia et al., 2016; Morrissette and Gubbels, 2020).

The centrosome is the main microtubule organizing center from which most basal bodies derive during flagellar assembly in other species. In *T. gondii* the centrosome of asexual stages consists of two 200 nm x 200 nm parallel centrioles with a 9 + 1 singlet microtubule geometry, a morphology that is notably distinct from canonical centrosomes and basal bodies (Dubremetz, 1971; Dubremetz, 1973; Morrissette and Sibley, 2002a; Francia and Striepen, 2014; Suvorova et al., 2015; Francia et al., 2016). Because the geometries and microtubule composition of the *T. gondii* basal body are ill-defined, it is still unclear whether it assembles from the asexual stage centrioles, or whether it is assembled *de novo*. *De novo* basal body synthesis has been proposed for *Plasmodium* sexual stages, as the asexual stages organize their asexual cell division through a “centriolar plaque” which consist of a few conserved components of other microtubule organizing centers, such as the centrosome, but lacks centrioles (Francia et al., 2016).

Canonical centriole biogenesis involves the hierarchical and sequential layout of structural components, catalyzed by overlapping regulatory signals in coordination with cell cycle progression. In metazoan, specialized kinases, such as PLK-4, Zyg1, or PLK1 trigger the centriole assembly cascade. An evolutionary conserved and ancestral protein module (known as UNIMOD; which includes SAS-6, SAS4/CPAP, and SAS5/STIL) whose occurrence is correlated with presence or loss of centrioles has been proposed to control centriole biogenesis, and to have emerged in the LECA (last eukaryotic common ancestor). However, homologs for the kinases, STIL, and other UNIMOD components, are not encoded for in the genome of *T. gondii*. Interestingly, the genome of *T. gondii* encodes for two SAS6 homologs (de Leon et al., 2013). SAS6 is a highly conserved and it is responsible for the cartwheel assembly, crucial for establishing centriole and basal body geometry. One of the homologs is found at the centrosome (TgSAS6) while the other one, named SAS6-Like (TgSAS6L), is located at the conoid, a structure proposed to have emerged from an ancestral MTOC whose original function was to nucleate flagella (Francia et al., 2012; de Leon et al., 2013; Back et al., 2020; O’Shaughnessy et al., 2020). Indeed, de Leon, and col., showed that a SAS6L homolog localizes to the basal body of the flagellated trypanosomatid, *Trypanosoma brucei*, leading them to propose that SAS6L could exhibit a similar localization in *T. gondii* (de Leon et al., 2013).

Canonical centriole biogenesis encompasses different isotypes of tubulins (involved in microtubule nucleation). Many of the homologs of these proteins were identified in *T. gondii*, while others are seemingly missing (Suvorova et al., 2015; Morlon-guyot et al., 2017). The presence of three α- and β-tubulin isotypes genes, δ- and ϵ-tubulin, SAS6 and SAS4 coding genes, indicate that this parasite could potentially assemble a canonical 9 + 0 triplet microtubule basal body (Nagel and Boothroyd, 1988; Garreau de Loubresse et al., 2001; Dupuis-Williams et al., 2002; Dutcher et al., 2002; Marshall and Rosenbaum, 2003; Xiao et al., 2010; Ross et al., 2013). In line with this, recent work by Ramakrishnan and col. found that the protein bearing the highest degree of similarity to ϵ-tubulin present in *T. gondii* genome, (originally annotated as β3-tubulin, but reclassified as ϵ-tubulin by Morlon-guyot et al., 2017), is indeed expressed in the enteroepithelial stages (Ramakrishnan et al., 2019).

*T. gondii* also encodes for a homolog of the centrosomal protein 164 (Cep164); homologs of this protein form appendages, a molecular signature that distinguishes the mother centriole from the daughter centriole in a basal body. Appendages serve to anchor the mother centriole to the membrane, enabling it to nucleate the flagellar axoneme. Though its role has not been explored, TgCEP164 could potentially be important for basal body anchoring (Morrissette, 2015; Morlon-guyot et al., 2017). Consistently, this protein does not express in aflagellated tachyzoites or bradyzoites as determined by asexual stages transcriptomic and proteomic experiments (Morlon-guyot et al., 2017). In contrast, evidence for TgCEP164 expression in enteroepithelial stages supports its functional role in sexual stages (Ramakrishnan et al., 2019).

Once flagella have been built, their maintenance requires several proteins. Intraflagellar Transport (IFT) is required to move structural and signaling components of the axoneme from the cell body to the tip and vice versa. IFTs and motor proteins such as kinesin and dynein coding genes can be identified in the *Toxoplasma gondii* genome (Farhat et al., 2020). However, their role in flagellar maintenance and parasite biology has not been explored.

### Where Am I? Molecular Cues Directing Gamete Formation Only in the Cat

The use of cats as experimental models has obvious ethical implications, it is costly and requires specialized animal housing facilities unavailable in the vast majority of research institutes. Notwithstanding, work done based on oocysts produced this way has been crucial in uncovering phenomena central to *T. gondii* dissemination and pathogenesis, including a plethora of structural studies (some, detailed above), as well as functional genomic and proteomics assays. Nonetheless, a long standing question in the field has been; why cats? In other words, what are the molecular cues that elicit the differentiation of *T. gondii* from bradyzoites into merozoites and then into gametocytes, exclusively at the cat’s intestinal epithelia. How are these signals interpreted and translated within the parasite?

Approaching this question has been technically challenging, as in principle it requires the use of companion animals as experimental models, and the temporal resolution of distinct stages is complicated by their overlap in *in vivo* infections. However, more cost-efficient, animal-friendly alternatives have been developed allowing researchers to dive into questions that were once inaccessible. One of such milestones has been the work done on enterocyte 2D cell cultures, and the generation of 3D enteroids, as models to study the interactions between the parasite and enterocytes (Moura et al., 2009; Luu et al., 2019). Though these approaches have greatly contributed, these can only partially replicate stage conversion.

More recently, an *in vitro* approach aimed at replicating the intestinal gut environment in 2D cultures has significantly contributed in this sense. Breakthrough work by Martorelli Di Genova and colleagues, approached a long-standing mystery in the field: what is the biochemical footprint that makes felines the selected definitive host? Briefly, the authors identified that host-specificity to felines in the sexual stage is owed to the accumulation of linoleic acid, given by the lack of delta-6-desaturase activity in their intestines, (Di Genova et al., 2019). Felines are the only mammals that lack this enzyme activity in their intestines. Remarkably, inhibiting this enzyme’s activity alone, and supplementing mice diet with an excess of linoleic acid, allowed them to now become *T. gondii* infectious oocyst spreaders (Di Genova et al., 2019).

The flip side of the question (i.e. what is happening within the parasite to elicit sexual differentiation)? was also recently approached and teased out. Farhat and colleagues found that a microrchidia homologue, named MORC1, assembles a complex with histone deacetylase 3 (HDAC3) and a number of apicomplexan transcription factors well known to influence stage transitions, known as ApiAP2s (Balaji et al., 2005; Farhat et al., 2020). MORC1 also regulates HAP2, a gene known to be required for macrogamete fertilization by the microgamete both in *T. gondii* and in *Plasmodium* (Liu et al., 2008; Angrisano et al., 2017; Ramakrishnan et al., 2019; Farhat et al., 2020). Interestingly HAP2 depletion leads to oocysts that are unable to sporulate. (Ramakrishnan et al., 2019).

Though formation of oocysts was not reported, conditional depletion of MORC1 was shown to elicit *in vitro* sexual differentiation by evidencing the formation of flagellar like structures, labeled by the expression of epitope tagged PF16, an armadillo repeat protein essential for flagellar function, in tissue culture. Therefore, MORC1 was put forward as a master regulator, controlling different transcriptional programs involved in stage transitions of sexual development (Farhat et al., 2020). Consistent with its proposed role, MORC1 depletion elicits gene expression of bradyzoite specific genes, 85% of the merozoite-specific transcripts, including those coding for proteins that are important for merozoite development like GRA11a,GRA11b, and merozoite-restricted surface proteins (SRS), as well as putative flagellar and basal body assembly proteins of the microgamete; six IFTs, nine kinesins, 11 dyneins, one basal body protein, and three radial spoke proteins (Farhat et al., 2020). Furthermore, MORC1 depletion derepresses the expression of 89% oocyst specific genes and almost all proteins present in fully sporulated oocyst (Farhat et al., 2020). MORC1 depletion also triggered expression of AP2IX-9, an ApiAP2 shown to prevent merozoites from switching back to bradyzoite, keeping the unidirectionality of the sexual stage development (Farhat et al., 2020). Downregulation of the tachyzoite invasion machinery was shown to accompany these processes (Hehl et al., 2015; Farhat et al., 2020).

## Closing Remarks

Understanding the biochemical footprint that made felines the selected host by *T. gondii* to complete its sexual differentiation constitutes a milestone in our comprehension on the biology of this parasite and a major technical breakthrough. The use of *in vitro* models for the study of sexual stages has opened the possibility for large scale molecular, dynamic and structural studies, avoiding the ethical considerations and technical limitations of using domestic animals as experimental models. No longer requiring the use of companion animals to quantitatively access sexual stages provides the scientific community with an unprecedented opportunity to dive into once unattainable biological questions relevant to persistence and dissemination such as those pertaining to basal body and flagellar assembly.

Understanding the molecular pathway leading up to committing to sexual stages is equally important, providing the potential of developing strategies to fight this parasite at the very origin of differentiation. These findings pave the way for generating genetically admixed strains *in vitro* opening new avenues for vaccine development, and new cell culture-based drug screening platforms for drugs aimed at sexual stages and the oocyst.

## Author Contributions

RT and MF wrote the manuscript and designed the Figure. All authors contributed to the article and approved the submitted version.

## Funding

RT and MF are funded by an installation grant by Banco de Seguros del Estado. The funder was not involved in the study design, collection, analysis, interpretation of data, the writing of this article or the decision to submit it for publication.

## Conflict of Interest

The authors declare that the research was conducted in the absence of any commercial or financial relationships that could be construed as a potential conflict of interest.
